# Supplementary material for: Improved predictions of phase behaviour of intrinsically disordered proteins by tuning the interaction range
Source: Open Res Eur. 2023 Jan 17;2:94. Originally published 2022 Aug 5. [Version 2] doi: 10.12688/openreseurope.14967.2 (PMC10450847; doi:10.12688/openreseurope.14967.2)
Supplement: Peer review history

# Peer review: Reviewer response for version 1

**DOI:** 10.21956/openreseurope.16180.r29852

**Published:** 2022-09-26

**Authors:** Frauke Gräter, Camilo Aponte-Santamaria

**Affiliations:** 1 Heidelberg Institute for Theoretical Studies (HITS), Heidelberg, Germany

Tesei and Lindorff-Larsen present CALVADOS 2 which is an improvement of CALVADOS 1, their original coarse-grained model to simulate the dynamics of intrinsically disordered protein chains or condensates of them.  They systematically studied the effect of the cutoff of the short-range interactions on the compactness of single chains and the phase behavior propensity.  They optimized the model using a large set of intrinsically disordered proteins of varying amino acid length and for which experimental data exist.

 This is a very important study as it shows to what extent the truncation of the short range interactions affect the dynamics of single chains and condensates of IDPs and how this feature can be used to balance the excess thermal energy needed to calibrate the original CALVADOS implementation.  

 I have the following comments.

 Similarly, as it was done for the short-range interactions, the electrostatic interactions are also truncated (at a cutoff distance of 4 nm which is about 4 fold the Debye length at 300 K and 150 mM ionic strength). I wonder what the influence of this cutoff is. The authors could comment on that.

 From equations 3 and 4, I understand that a temperature-dependent Debye length was used. How much does this length change when changing the temperature (relative to the cutoff)? I think it is important to comment on that in the paper.  

 Minor points:

- what are the black lines in Fig 3B?
- Intro 2nd paragraph:  What is M1? M1 has not been defined.
- Methods:  simulation length of 6*0.3*N^2 ps. What was the motivation to choose this particular simulation length?
- Fig 4B, orange-yaxis: title confusing. It is not the normalized temperature ratio but the kinetic energy ratio.

Is the study design appropriate and does the work have academic merit?

Yes

Is the work clearly and accurately presented and does it cite the current literature?

Yes

If applicable, is the statistical analysis and its interpretation appropriate?

Yes

Are all the source data underlying the results available to ensure full reproducibility?

Yes

Are the conclusions drawn adequately supported by the results?

Yes

Are sufficient details of methods and analysis provided to allow replication by others?

Yes

Reviewer Expertise:

Molecular Dynamics, IDPs, coarse-graining

We confirm that we have read this submission and believe that we have an appropriate level of expertise to confirm that it is of an acceptable scientific standard.

# Response

**Published:** 2022-12-14

**Authors:** Giulio Tesei

We thank Frauke Gräter and Camilo Aponte-Santamaria for their comments and positive view on our work.

 Below is our point-by-point response to the comments:

- *Similarly, as it was done for the short-range interactions, the electrostatic interactions are also truncated (at a cutoff distance of 4 nm which is about 4 fold the Debye length at 300 K and 150 mM ionic strength). I wonder what the influence of this cutoff is. The authors could comment on that.*
**Response**: ****We agree with the reviewers that the effect of the cutoff on the ionic interactions is an interesting aspect to examine. We first tested the effect on the single chain data. Although some proteins (A2 LCD and CAHSD) were simulated at low ionic strength ( *c _s _* = 5–70 mM), we found that increasing the cutoff value from 4 to 6 nm leads to relative changes in the predicted *R _g_* values below 2.5% (Figure S8A). However, the effect on the predicted PRE data is more pronounced. In particular, for A2 LCD we observe a ~30% decrease in the *X* ^2^_PRE_ value (Figure S8B).
  We also tested the effect on the phase behavior of A2 LCD ( *c _s _* = 10 mM), A1 LCD* ( *c _s _* = 150 mM), FUS LCD ( *c _s _* = 150 mM), LAF-1 RGG domain ( *c _s _* = 150 mM), and its shuffled variant ( *c _s _* = 150 mM). As expected, increasing the cutoff from 4 to 6 nm has a negligible effect on the phase separation at high *c _s _* whereas a considerable increase in saturation concentration, *c _sat_*, is observed for simulations of A2 LCD at *c _s _* = 10 mM (Figure S8C). Coincidentally, we found that the *c _sat_* predicted for A2 LCD using the shorter cutoff is in better agreement with the reference experimental value. We now discuss this point in the results section: In the model, ionic interactions are also truncated and shifted. At the cutoff distance of 4 nm, the ionic energy decreases with increasing salt concentration and amounts to ±2.7 J mol ^-1^ at *c _s _* = 150 mM and 20 ºC. However, this energy is ~43 times larger at *c _s _* = 10 mM, which suggests that the model may considerably underestimate the strength and range of charge-charge interactions at low salt concentrations. To investigate this aspect, we performed single-chain and direct-coexistence simulations using a longer cutoff of 6 nm for the ionic interactions. The change in cutoff has a small effect on both the *R _g_* (Figure S8A) and the *c _sat_* values predicted for systems at *c _s _* = 150 mM (Figure S8C). Conversely, simulations at low salt concentration are considerably affected by the increase in cutoff. For the PRE data of A2 LCD at *c _s _* = 5 mM, we observe an improvement in the agreement with experiments (Figure S8B). Instead, the accuracy of the phase behaviour predicted for A2 LCD at *c _s _* = 10 mM decreases significantly as the *c _sat_* value shows a ~100-fold increase (Figure S8C). Since the vast majority of the available *R _g_* and *c _sat_* data in our training and test sets was measured at *c _s_* ≈150 mM, we are currently unable to further assess or improve the accuracy of the model at low salt concentrations.
- *From equations 3 and 4, I understand that a temperature-dependent Debye length was used. How much does this length change when changing the temperature (relative to the cutoff)? I think it is important to comment on that in the paper.  *
**Response**: The Debye length, *D*, used in the model shows a weak temperature dependence. For example, the relative change in *D* upon an increase in temperature from 4 to 50 ºC is only -3%. Ionic interaction energies between like-charge residues at a cutoff distance of 4 nm, *u _DH_* ( *r *= 4 nm), also show a weak temperature dependence. At *c _s _* = 150 mM, *u _DH_* ( *r *= 4 nm) is 2.6 J mol ^-1^ at at 4 ºC and 2.8 J mol ^-1^ at 50 ºC. We now discuss this point in the methods section: As previously observed [doi:10.1038/ s43588-021-00155-3], accounting for the temperature-dependence of *E*_*r*_ has a small effect on the predictions of the model. Indeed, the relative change in *D* upon an increase in temperature from 4 to 50 ºC is only -3%. Similarly, at *c _s _* = 150 mM, the Debye-Hückel energy between like-charged residues at the cutoff distance, *u _DH_* ( *r *= 4 nm), is 2.6 J mol ^-1^ at at 4 ºC and 2.8 J mol ^-1^ at 50 ºC.
- *What are the black lines in Fig 3B?*
**Response**: We have clarified in the figure caption that the black error bars represent the relative error of the experimental measurement, i.e. *σ ^exp^ / R _g_^exp^*.
- *Intro 2nd paragraph:  What is M1? M1 has not been defined.*
**Response**: We have changed the text to specify that M1 is the name used to refer to the CALVADOS 1 optimal stickiness parameters in our previous publication on the model (Tesei et al. DOI: 10.1073/pnas.2111696118).
- *Methods: simulation length of 6 x 0.3 x N ^2^ ps. What was the motivation to choose this particular simulation length?*
**Response**: We have changed the text to clarify how we chose sampling frequencies and simulation lengths based on sequence length, *N*. Briefly, we saved every **Δ** *t ≈ 3 x N ^2^* fs if *N>100*, and **Δ** *t *= 30 ps otherwise, to ensure that the radii of gyration for consecutive frames were weakly correlated irrespective of sequence length, *N* (Figure 1). The quadratic *N*-dependence of **​​​​​​​Δ** *t * was inferred from the lag time at which the autocorrelation function of the *R _g_* approximates 1/2, for a subset of proteins of different *N*. We simulated ten replicas per sequence, each for a simulation time of *600 x* **​​​​​​​Δ** *t *. After discarding the initial 100 frames of each replica, we obtained 5,000 weakly correlated conformations for each protein. In the context of the optimization procedure, we observed that this number of frames is sufficient for accurately reweighting the trajectories, when the fractions of effective frames exceeds 60%.
  ​​​​​​​
- *Fig 4B, orange y-axis: title confusing. It is not the normalized temperature ratio but the kinetic energy ratio.*
**Response**: We have clarified in the caption that the orange y-axis shows the ratio of thermal energies, *RT' / RT*, where *R* is the gas constant.

# Peer review: Reviewer response for version 1

**DOI:** 10.21956/openreseurope.16180.r29851

**Published:** 2022-09-02

**Authors:** Alex Holehouse

**Affiliations:** 1 Department of Biochemistry and Molecular Biophysics, Washington University School of Medicine, St. Louis, MO, USA

This paper is a timely update to the recent CALVADOS forcefield developed by Tesei *et al.* and published late last year. The authors demonstrate how while tweaking aspects of CALVDOS do not substantially alter the single-chain radii of gyrations, the phase behavior can be altered substantially.

 The updated version provides some interesting discussion, the data are all shared on GitHub and Zenodo, and all software parameters are made available in a convenient format. The paper is well-written, the methods well-described and logically motivated and the figures clear. 

 What more could one want from a paper? I strongly support indexing in its current form.

 As a note, the conciseness of this review should not be seen as a lack of attention to detail. However, would any suggestions or comments I make materially affect the conclusions, clarity, or availability of data? I do not think so, and as such, it is in the author's best interest to use their time on the next set of questions than fine-tune what is already a very strong manuscript.

Is the study design appropriate and does the work have academic merit?

Yes

Is the work clearly and accurately presented and does it cite the current literature?

Yes

If applicable, is the statistical analysis and its interpretation appropriate?

Yes

Are all the source data underlying the results available to ensure full reproducibility?

Yes

Are the conclusions drawn adequately supported by the results?

Yes

Are sufficient details of methods and analysis provided to allow replication by others?

Yes

Reviewer Expertise:

Coarse-grained simulations of disordered proteins

I confirm that I have read this submission and believe that I have an appropriate level of expertise to confirm that it is of an acceptable scientific standard.

# Response

**Published:** 2022-12-14

**Authors:** Giulio Tesei

We thank Alex Holehouse for the very kind comments and positive view on our work.
